# Synergistic PM_2.5_ and O_3_ control to address the emerging global PM_2.5_-O_3_ compound pollution challenges

**DOI:** 10.1016/j.eehl.2024.04.004

**Published:** 2024-04-19

**Authors:** Chao He, Jianhua Liu, Yiqi Zhou, Jingwei Zhou, Lu Zhang, Yifei Wang, Lu Liu, Sha Peng

**Affiliations:** a College of Resources and Environment, Yangtze University, Wuhan 430100, China; b Hubei Key Laboratory of Petroleum Geochemistry and Environment, Yangtze University, Wuhan 430100, China; c School of Geography and Ocean Science, Nanjing University, Nanjing 210023, China; d Hydrology and Environmental Hydraulics Group, Wageningen University and Research, Wageningen 6700 HB, the Netherlands; e State Key Laboratory of Freshwater Ecology and Biotechnology, Institute of Hydrobiology, Chinese Academy of Sciences, Wuhan 430072, China; f State Key Joint Laboratory for Environmental Simulation and Pollution Control, School of Environmental Sciences and Engineering, Peking University, Beijing 100871, China; g State Key Laboratory of Pollution Control and Resource Reuse, School of Environment, Nanjing University, Nanjing 210023, China; h Collaborative Innovation Center for Emissions Trading System Co-constructed by the Province and Ministry, Hubei University of Economics, Wuhan 430205, China

**Keywords:** PM_2.5_-O_3_ compound pollution, Population exposure risk, Spatial correlation, Synergistic treatment potential

## Abstract

In recent years, the issue of PM_2.5_-O_3_ compound pollution has become a significant global environmental concern. This study examines the spatial and temporal patterns of global PM_2.5_-O_3_ compound pollution and exposure risks, firstly at the global and urban scale, using spatial statistical regression, exposure risk assessment, and trend analyses based on the datasets of daily PM_2.5_ and surface O_3_ concentrations monitored in 120 cities around the world from 2019 to 2022. Additionally, on the basis of the common emission sources, spatial heterogeneity, interacting chemical mechanisms, and synergistic exposure risk levels between PM_2.5_ and O_3_ pollution, we proposed a synergistic PM_2.5_-O_3_ control framework for the joint control of PM_2.5_ and O_3_. The results indicated that: (1) Nearly 50% of cities worldwide were affected by PM_2.5_-O_3_ compound pollution, with China, South Korea, Japan, and India being the global hotspots for PM_2.5_-O_3_ compound pollution; (2) Cities with PM_2.5_-O_3_ compound pollution have exposure risk levels dominated by ST + ST (Stabilization) and ST + HR (High Risk). Exposure risk levels of compound pollution in developing countries are significantly higher than those in developed countries, with unequal exposure characteristics; (3) The selected cities showed significant positive spatial correlations between PM_2.5_ and O_3_ concentrations, which were consistent with the spatial distribution of the precursors NOx and VOCs; (4) During the study period, 52.5% of cities worldwide achieved synergistic reductions in annual average PM_2.5_ and O_3_ concentrations. The average PM_2.5_ concentration in these cities decreased by 13.97%, while the average O_3_ concentration decreased by 19.18%. This new solution offers the opportunity to construct intelligent and healthy cities in the upcoming low–carbon transition.

## Highlights

• 47.5% of assessed cities worldwide are exposed to compound PM_2.5_-O_3_ pollution.
• Cities in developing countries experience higher exposure risks than developed countries.
• Significant positive spatial correlations between PM_2.5_ and O_3_ concentrations are observed.
• 52.5% of cities worldwide have the potential for synergistic PM_2.5_ and O_3_ reductions.

## Graphical abstract

**Figure:**
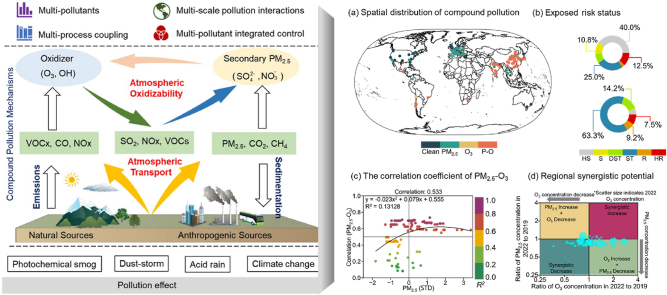

## 1 Introduction

Elevated concentrations of fine particulate matter (PM_2.5_) and surface ozone (O_3_) are harmful to human health [1,2], ecosystems [3], and crop yields [4,5], and are a major contributor to climate change [6,7]. PM_2.5_ is composed of directly emitted primary PM_2.5_ and secondary PM_2.5_, which is formed from gaseous precursors, including SO_2_, nitrogen oxides (NOx), volatile organic compounds (VOCs), and NH_3_ [8]. O_3_ generation, beyond that originating from stratospheric transport, primarily occurs through complex photochemical reactions between NOx and VOCs under sunlight [9]. Recent collaborative efforts by the World Health Organization (WHO) and global governments have led to a notable reduction in PM_2.5_ concentrations worldwide, particularly in certain cities within affluent European and North American nations, where levels have approached or met the WHO's IT-1 target value of 35 μg/m^3^ [10]. However, according to data from the 2019 “Global Air Status Report” (https://www.stateofglobalair.org/), 54% of the global population lives in areas above the 35 μg/m^3^ threshold, resulting in approximately 2.9 million premature deaths from PM_2.5_ exposure. Concurrently, there is growing evidence that global O_3_ pollution is becoming more visible, with a wider range of impacts and longer pollution season [11]. According to the Global Burden of Disease (GBD), weighted O_3_ concentrations in 11 populous nations range from 45 to 68 ppb, approaching or exceeding the WHO guideline of 100 μg/m^3^. In 2019 alone, O_3_ exposure resulted in 365,000 premature deaths worldwide [12]. Amid this context, studies have shown that the health hazards of global air pollution will become more severe in the future, driven by climate change, and that the features of pollution have shifted from single soot-type pollution in the past to compound atmospheric pollution with multiple sources of emissions and multiple pollutants coexisting and interacting with each other [13]. Therefore, clarifying the issue of PM_2.5_ and O_3_ compound pollution has become an important atmospheric environmental issue for the next step of improving air quality and realizing environmental sustainability processes globally.

To effectively combat the global pollution caused by PM_2.5_ and O_3_ compounds, it is crucial to accurately identify the current challenges, gain knowledge from historical experiences of PM_2.5_ and O_3_ pollution management, and ultimately construct a synergistic control framework for PM_2.5_ and O_3_ pollution. Recognized as a global menace, scholars have rigorously examined PM_2.5_ and O_3_ pollution across diverse spatial scales, delving into their spatiotemporal distribution [14], regional transport mechanisms [15], chemical mechanisms [16,17], drivers [18,19], economic ramifications [20], and health implications [21]. For instance, Zhao et al. [22] examined the worldwide spatial and temporal trends and population exposure risk of PM_2.5_ concentrations from 2000 to 2016, clarifying the relationship between PM_2.5_ concentrations and population exposure risk. From a spatiotemporal lens, Lim et al. [23] identified principal socio-economic elements shaping the spatial alterations in global PM_2.5_ concentrations, subsequently proposing mitigation pathways tailored to nations' economic standings. Approaching from a sustainability perspective, Zhou et al. [24] explored the spatiotemporal trends and population exposure risk of global springtime O_3_ concentrations, pinpointing pivotal meteorological determinants influencing different regional O_3_ fluctuations and associated human risks. Further, studies by Zhang et al. [25] and Lyu et al. [26] provided comprehensive insights into the health hazards and climate impacts linked to global O_3_ pollution.

Concurrently, a plethora of studies have identified a regional synergy in the pollution patterns of PM_2.5_ and O_3_. This synergistic feature has been universally observed across cities globally [27]. For instance, Zhao et al. [28] examined the spatiotemporal association of PM_2.5_ and O_3_ pollution in 367 key cities in China from 2015 to 2019. Their findings highlighted that those regions with the most severe PM_2.5_ pollution concurrently suffered from intense O_3_ pollution. In a similar vein, Sicard et al. [29] scrutinized the interplay between PM_2.5_ and O_3_ during air pollution episodes in arid continental climates based on air quality data from 21 ground monitoring stations in the Middle East. They discerned that whenever PM_2.5_ concentrations surged, a concurrent oscillation in O_3_ concentrations was evident. Analogous phenomena have been documented in the US [30] and Europe [31,32] through multi-year air quality monitoring. Moreover, burgeoning evidence posits that PM_2.5_ and O_3_ share common precursors, with VOCs and NOx emerging as their most pivotal shared antecedents [33]. On one hand, NOx and VOCs influence PM_2.5_ concentrations by fostering the formation of nitrates and secondary organic aerosols, and simultaneously play a significant role in the chemistry of O_3_. On the other hand, the heterogeneous reactions on the surface of particulate matter can directly adsorb O_3_ or react with nitrogen oxides (NO_2_, NO_3_, N_2_O_5_), thereby affecting O_3_ concentration [34]. Specifically, from 2000 to 2019, there was a slight global decrease in PM_2.5_ exposure (on average, −0.2% per year). However, 65% of cities still showed an increasing trend in PM_2.5_ exposure levels. Additionally, the O_3_ exposure levels of the global urban population increased (on average, +0.8% per year) due to the reduced titration effect of NO on ozone [35]. Even at night, O_3_ levels continued to rise [36]. This shared origin trait of PM_2.5_ and O_3_ has been ubiquitously recognized globally. Therefore, coordinated control of PM_2.5_ and O_3_ compound pollution from the perspective of synergistic regional emissions and the same sources of PM_2.5_ and O_3_ has become the key to managing global compound pollution.

Facing the escalating global challenge of PM_2.5_ and O_3_ compound pollution, scholars have embarked on extensive research to elucidate the characteristics of pollution, driving factors, and underlying mechanisms, aiming to devise collaborative mitigation strategies. Such endeavors aspire to offer technical support for the continuous improvement of air quality and public health protection across diverse regions globally. For instance, Wang et al. [37] probed into the causality of PM_2.5_ and O_3_ compounded pollution from the perspective of active nitrogen transformation routes in atmospheric nitrogen cycling. Dai et al. [38], leveraging a refined emission inventory of the Yangtze River Delta in China and the WRF-CMAQ model, crafted synergistic control pathways for atmospheric PM_2.5_ and ozone pollution in the region. Ojha et al. [19] reviewed mechanisms and methods for the collaborative control of PM_2.5_ and O_3_, positioning it within the context of global warming. Meanwhile, Faridi et al. [39] furnished a comprehensive assessment of long-term trends and health implications of PM_2.5_ and O_3_ pollution in Tehran, grounded on real-time hourly concentration datasets from 21 air quality monitoring stations spanning 2006–2015. Such studies grant a pivotal theoretical foundation and empirical insight into the driving forces behind air pollution in various global regions. Nonetheless, there remain gaps in this arena. Historically, many studies gravitated towards analyzing a particular air pollutant, with scant research addressing the spatiotemporal correlation features of compounded pollutants, let alone delving into their intricate interrelations. Further, due to a dearth of pollutant concentration data, assessing the spatiotemporal evolution of pollutants on a global scale remains a challenge. Most critically, there's a conspicuous absence of research offering a holistic understanding of PM_2.5_ and O_3_ compounded pollution traits from a global viewpoint, especially within a sustainable development lens that evaluates exposure risks to populations. Concurrently, no framework has been discerned thus far that addresses the collaborative governance of global PM_2.5_ and O_3_ compounded pollution.

To address the identified knowledge gaps, this study utilizes PM_2.5_ and O_3_ concentration monitoring data from 120 cities globally spanning from 2019 to 2022. Leveraging methodologies such as spatial statistical analysis, time series analysis, exposure risk assessment, and spatial correlation analysis, this research represents the first comprehensive global-scale investigation into the spatiotemporal patterns, evolutionary characteristics, exposure risks, and spatial associations with precursor substances of combined PM_2.5_ and O_3_ pollution. This work deepens our understanding of the concurrent management of PM_2.5_ and O_3_ on a global scale, proposing an integrated framework for their co-management. The findings stand to foster collaboration between the air quality and climate communities, offering policymakers crucial insights to jointly address these persistently intertwined threats.

## 2 Materials and methods

### 2.1 Study area

**Fig. 1:**
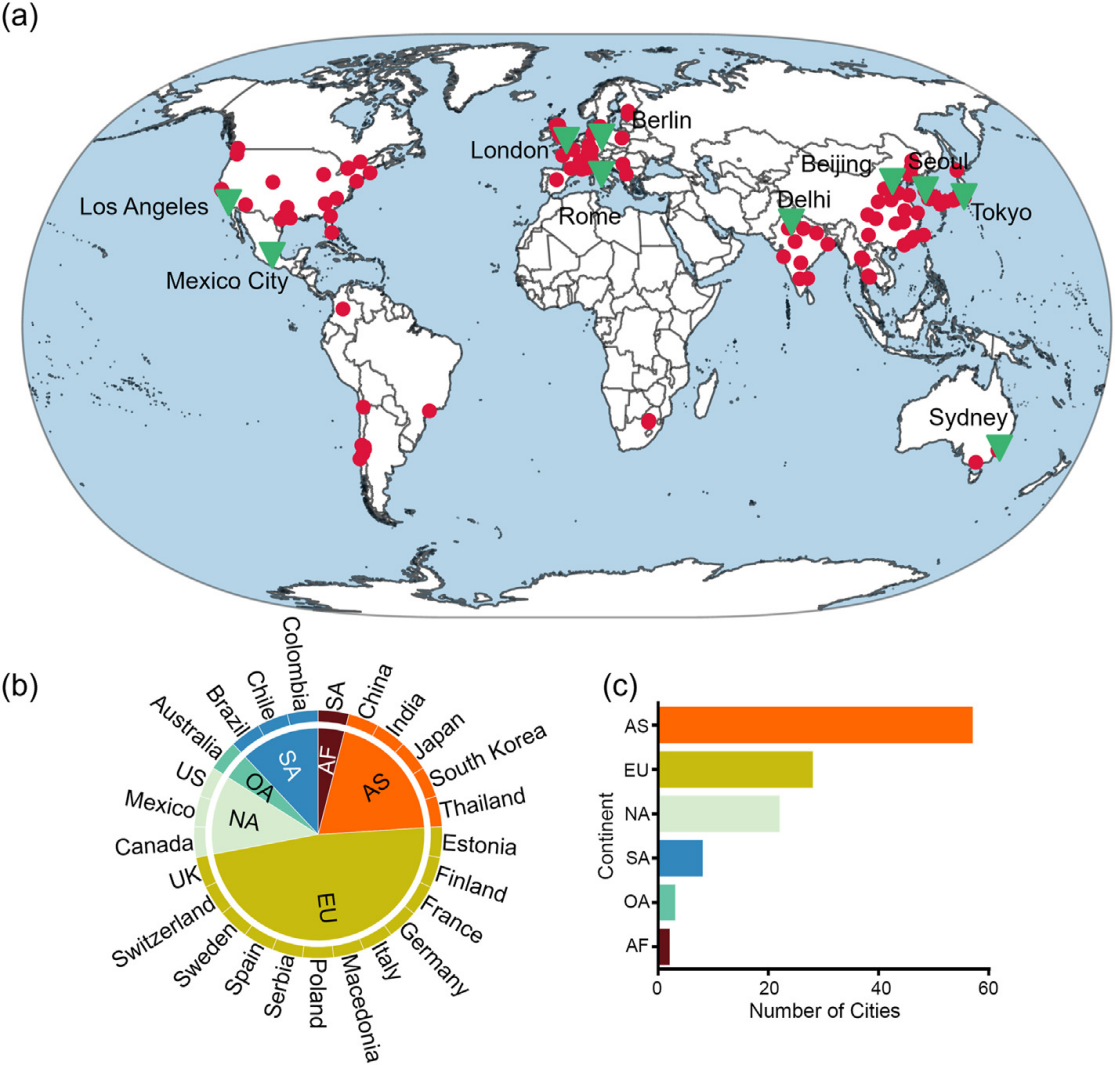
Spatial distribution of the study areas. The red dots represent the selected 120 cities, while the green triangles indicate the 10 focal cities (a). The pie chart displays the number of countries from each continent (b), and the bar chart shows the number of cities included from each continent (c).

In this study, we focus on 120 major cities worldwide. These cities are primarily located in Asia (57), Europe (28), North America (22), South America (8), Oceania (3), and Africa (2). The primary reasons for selecting these cities are as follows: Firstly, the chosen cities have high population densities, high anthropogenic emissions, high energy consumption, and elevated levels of air pollution [[40], [41], [42], [43]]. Secondly, data from these cities possess a complete time series, allowing for quantitative analyses over various temporal scales. Lastly, these cities have diverse geographical and climatic conditions. For instance, Beijing has a temperate monsoon climate, while Delhi has a semi-arid climate. These varying geographical and climatic conditions are crucial in enhancing our comprehension of the spatial heterogeneity of PM_2.5_ and O_3_ compound pollution. Given these facts, the chosen cities offer a suitable variety of diverse regions for our investigation. Moreover, to delve deeper into the compound pollution status of PM_2.5_ and O_3_ at the urban scale, we selected 10 cities out of the 120, namely Beijing (China), Tokyo (Japan), Seoul (South Korea), Delhi (India), Sydney (Australia), London (UK), Rome (Italy), Berlin (Germany), Los Angeles (US), and Mexico City (Mexico) for in-depth analysis. The spatial distribution of the 120 cities and the 10 focus cities is illustrated in Fig. 1.

### 2.2 Data sources and preprocessing

The daily records of PM_2.5_ and O_3_ concentrations across the 120 chosen cities were sourced from the World Air Quality Index (WAQI) portal (https://www.aqicn.org/). To analyze the co-sourced features of PM_2.5_ and O_3_ and their effect on the exposure risk of the population, we collected precursor emission inventories (VOCs, NOx) and population inventories from the European Commission (https://commission.europa.eu/) and The World Bank (https://www.worldbank.org/), respectively. Prior to conducting any analysis, based on the study by He et al. [27], we implemented data quality control measures on the daily PM_2.5_ and O_3_ concentrations obtained from 120 cities globally. We discarded anomalous data that did not meet the statistical criteria, such as daily PM_2.5_ and O_3_ concentrations that exceeded 999 μg/m^3^. In this study, the valid counting days for monthly and annual average concentrations of PM_2.5_ and O_3_ in cities are no less than 27 days and 360 days, respectively. Concurrently, this study evaluated the risk of exposure to PM_2.5_ and O_3_ pollution with reference to the new Air Quality Guidelines (AQG) issued by the World Health Organization in 2021 [44]. In the specific calculations, we utilized the rolling average of the maximum 8-h concentrations as the daily average concentration for O_3_.

### 2.3 Definition of PM_2.5_ and O_3_ compound pollution

Drawing from past epidemiological studies on the population exposure to PM_2.5_ and O_3_ [45,46] and the new AQG standards, we have chosen the daily average concentrations of PM_2.5_ and O_3_ to be 35 μg/m^3^ and 100 μg/m^3^, respectively, as the thresholds for categorizing the dominant pollution types of PM_2.5_ and O_3_. Based on this scheme, we classify the dominant pollution types of PM_2.5_ and O_3_ into the following four categories: Compound Pollution of PM_2.5_ and O_3_ (P–O), PM_2.5_ Dominant Pollution, O_3_ Dominant Pollution, and Clean. The detailed categorization criteria are illustrated in Table 1.

**Table 1:** Compound Pollution Classification Standards.

PM_2.5_ (μg/m^3^)	O_3_ (μg/m^3^)	Pollution dominant type
ρ(PM_2.5_) > 35	ρ(O_3_) > 100	P–O
ρ(PM_2.5_) > 35	ρ(O_3_) < 100	PM_2.5_ dominated pollution
ρ(PM_2.5_) < 35	ρ(O_3_) > 100	O_3_ dominated pollution
ρ(PM_2.5_) < 35	ρ(O_3_) < 100	Clean

### 2.4 Exposure risk assessment of compound pollution

This study discusses the risk of population exposure to long-term ambient PM_2.5_ and O_3_ based on the method by Lim et al. [23]. Initially, we employed the Mann-Kendall method [47,48] to analyze the changing trends of PM_2.5_ and O_3_ concentrations over the research period. The calculations for the Mann-Kendall method are as given in Equations (1), (2), (3), (4):

(1) $$\begin{array}{c} S=\sum_{i=1}^{n-1}\sum_{j=i+1}^{n}sgn\left(x_{j}-x_{i}\right)\; \end{array}$$

where: *n* represents the total number of data points; *X*_*i*_ and *X*_*j*_ are data values in time series *i* and *j*. *X*_*j*_ is used as a reference point to compare with the remaining data points *X*_*i*_. The *sgn(x*_*j*_*-x*_*i*_*)* is the sign function, with the specific formula as follows:

(2) $$\begin{array}{c} sgn\left(x_{j}-x_{i}\right)=\left\{ \begin{array}{c} \begin{array}{cc} +1, & x_{j}-x_{i}>0 \end{array}\\ \begin{array}{cc} 0, & x_{j}-x_{i}=0 \end{array}\\ \begin{array}{cc} -1, & x_{j}-x_{i}<0 \end{array} \end{array}\right.\; \end{array}$$

Additionally, the formula for calculating the variance is:

(3) $$\begin{array}{c} Var\left(S\right)=\frac{1}{18}\left[n\left(n-1\right)\left(2n+5\right)-\sum_{k=1}^{p}q_{k}\left(q_{k}-1\right)\left(2q_{k}+5\right)\right]\; \end{array}$$

In the formula, *n* represents the total number of data points; *p* denotes the number of tied groups; *q*_*k*_ indicates the number of data points contained in the *k*-th tied group. When dealing with large samples (*n* > 10), the standardized test statistic *Z* is used for calculations:

(4) $$\begin{array}{c} Z=\left\{ \begin{array}{cc} \frac{S-1}{\sqrt{Var\left(S\right)}}, & S>0\\ 0, & S=0\\ \frac{S+1}{\sqrt{Var\left(S\right)}}, & S<0 \end{array}\right.\; \end{array}$$

By evaluating the *Z* value, a statistically significant curve trend can be obtained. A positive *Z* indicates an increasing trend, while a negative *Z* indicates a decreasing trend. In a two-tailed trend test, for a given confidence level (significance level) *α*, if |*Z*| ≥ *Z*_*1-α/2*_, then the null hypothesis *H*_*0*_ is rejected. This means that, at the confidence level *α*, the time series data exhibits a significant increasing or decreasing trend. |*Z*| values greater than or equal to 1.645, 1.960, and 2.576 represent passing the significance test at confidence levels of 90%, 95%, and 99%, respectively.

Subsequently, combining the high or low levels and change trends of PM_2.5_ or O_3_ concentrations, we classified the exposure risk level of the population in different cities under the PM_2.5_ and O_3_ environments into six types: High Risk (HR), Stabilization (ST), Risk (R), Deep Stabilization (DST), Safety (S), and High Safety (HS). Among them, HR and ST both indicate extremely high pollutant concentrations, but HR denotes an increasing trend in pollutant concentration, while ST signifies a decreasing trend. R and DST mean high pollutant concentrations with respective increasing and decreasing trends. S and HS indicate low pollutant concentrations, with respective increasing and decreasing trends. Here, based on the epidemiological methods in Strak et al. [49], Guan et al. [45], and Guerreiro et al. [46], we define extremely high pollutant concentration criteria as ρ(PM_2.5_) > 35 μg/m^3^ or ρ(O_3_) > 120 μg/m^3^; High pollutant concentration criteria as 25 μg/m^3^ < ρ(PM_2.5_) < 35 μg/m^3^ or 100 μg/m^3^ < ρ(O_3_) <120 μg/m^3^; Low pollutant concentration criteria as ρ(PM_2.5_) < 25 μg/m^3^ or ρ(O_3_) < 100 μg/m^3^.

### 2.5 Spatial correlation analysis

In this study, Bivariate Moran's I (Bi-Moran's I), spatial statistical analysis, and spatial correlation analysis models were employed to investigate the spatial agglomeration characteristics, spatial correlations, and spatial associations with the main precursors (NOx and VOCs) of PM_2.5_ and O_3_ concentrations in the 120 global cities during the study period. The calculation for the Bi-Moran's I is as per Equation 5:

(5) $$\begin{array}{c} I_{i}^{B}=cx_{i}\sum_{j}w_{ij}y_{j}\; \end{array}$$

In the formula, $I_{i}^{B}$ represents the bivariate local Moran's index for region *i*; *w*_*ij*_ is an element of the spatial weight matrix; and *c* is a constant proportionality factor. This index is used to quantitatively describe the degree of association between variable *x* in region *i* and variable *y* in neighboring region *j*. Furthermore, the detailed calculation process of the spatial correlation analysis model can be found in the research by Lu et al. [50]. The spatial analyses and implementation involved in this study are primarily conducted using the GeoDa1.20 (http://geodacenter.github.io/), ArcGIS10.7 (https://www.esri.com/), and GWmodelS1.0.3 (https://github.com/GWmodel-Lab/GWmodelS/) software.

### 2.6 Analysis of synergistic changes in compound pollution

In this study, we measure the level of synergistic changes in PM_2.5_ and O_3_ concentrations based on the relative rate of change (*ROC*) of PM_2.5_ and O_3_ concentrations in 2019 and 2022, calculated as in Equation 6:

(6) $$\begin{array}{c} If=\left\{ \begin{array}{cc} {ROC}_{{i,PM}_{2.5}}\geq 1\;and\;{ROC}_{i,O_{3}}\geq 1, & Synergistic\;Increase\\ {ROC}_{{i,PM}_{2.5}}\;<1\;and\;{ROC}_{i,O_{3}}<1, & Synergistic\;Decrease\\ {ROC}_{{i,PM}_{2.5}}\geq 1\;and\;{ROC}_{i,O_{3}}<1, & {PM}_{2.5}\;Increase\;and\;O_{3}\;Decrease\\ {ROC}_{{i,PM}_{2.5}}\;<1\;and\;{ROC}_{i,O_{3}}\geq 1, & {PM}_{2.5}\;Decrease\;and\;O_{3}\;Increase \end{array}\right.\; \end{array}$$

The *ROC_i_* in equation is calculated as follows:

(7) $$\begin{array}{c} {ROC}_{i}=\frac{C_{i,2022}}{C_{i,2019}}\; \end{array}$$

where: *ROC* represents the relative change of PM_2.5_ and O_3_ in city *i*; *C*_*i,2022*_ and *C*_*i,2019*_ represent the concentrations of PM_2.5_ and O_3_ in city *i* in 2022 and 2019, respectively.

## 3 Results

### 3.1 Temporal and spatial distribution of global PM_2.5_ and O_3_ concentrations

**Fig. 2:**
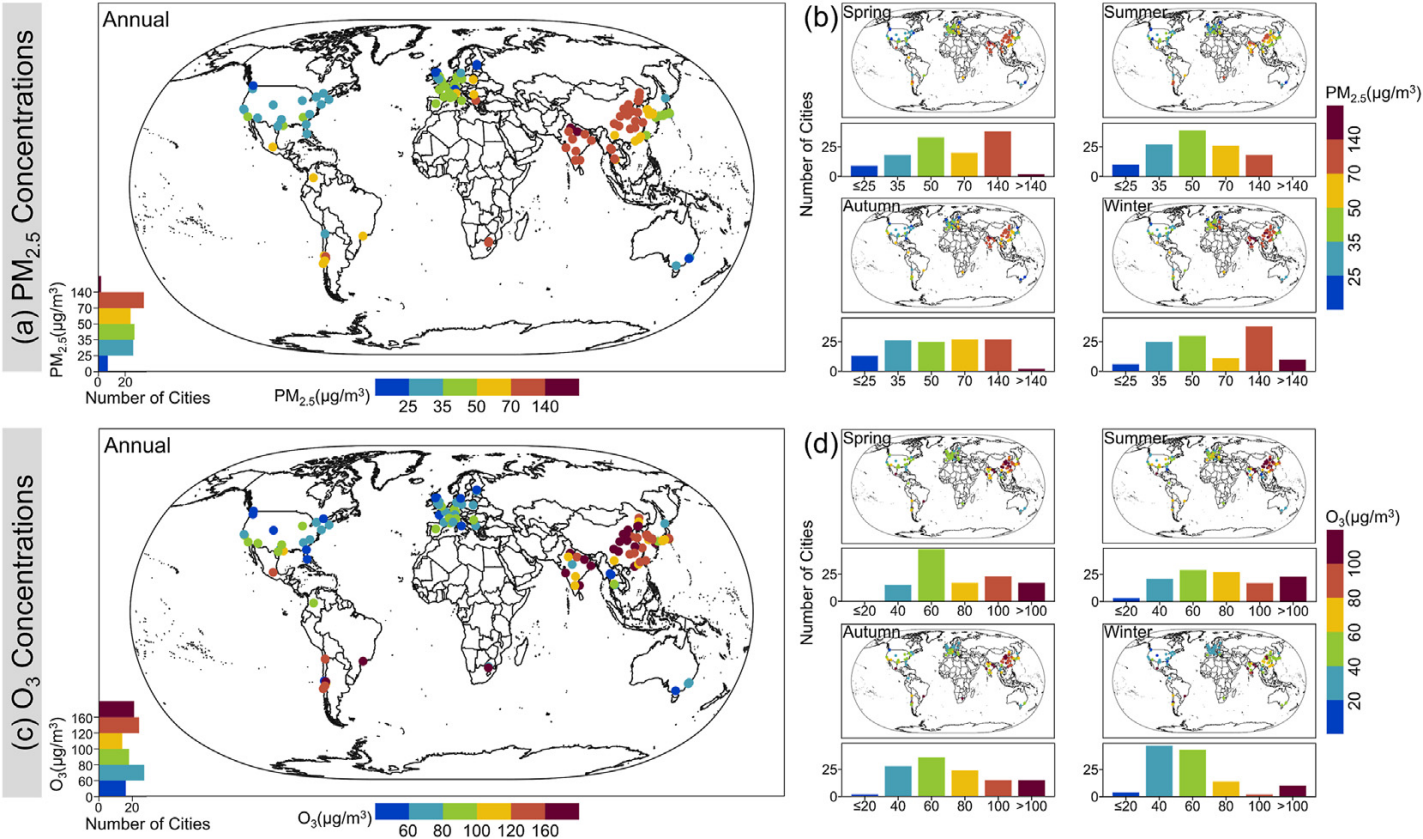
The spatial distribution of annual average PM_2.5_ and O_3_ concentrations in 120 cities globally from 2019 to 2022 (a and c), and the spatiotemporal distribution of PM_2.5_ and O_3_ concentrations on a seasonal basis (b and d). The bar chart indicates the number of cities exposed to various PM_2.5_ and O_3_ concentration levels.

Fig. 2a and b depicts the spatial distribution and seasonal variations of the annual average PM_2.5_ concentrations in 120 global cities from 2019 to 2022. The annual average PM_2.5_ concentrations for these cities from 2019 to 2022 were 61.86, 56.92, 57.36, and 55.48 μg/m^3^, respectively, indicating a fluctuating downward trend. Among the selected cities, less than 1% were found to have low PM_2.5_ exposures (4-year average PM_2.5_ concentrations ≤ 25 μg/m^3^). These cities are primarily situated in Canada, Australia, and several countries in Europe, such as Vancouver (Canada, 18.46 μg/m^3^), Wollongong (Australia, 22.17 μg/m^3^), and Edinburgh (UK, 20.56 μg/m^3^). In contrast, high PM_2.5_ concentrations were found in 30% of the cities, where the average concentration of PM_2.5_ over four years exceeded 70 μg/m^3^. These cities are mainly found in eastern China, northern and southwestern India, and also include Santiago (Chile, 75.37 μg/m^3^) and Johannesburg (South Africa, 75.77 μg/m^3^). Notably, northern Indian cities such as Lucknow (146.5 μg/m^3^) and Delhi (161.9 μg/m^3^) registered 4-year average PM_2.5_ concentrations exceeding 140 μg/m^3^. Meanwhile, over 40% of cities had exposures to 4-year average PM_2.5_ concentrations ranging from 35 to 70 μg/m^3^, predominantly located in countries such as South Korea, Japan, France, the UK, and Germany. It was observed that the global PM_2.5_ concentrations were at their zenith during the winter and at a nadir during the summer. Compared to summer, the number of cities exposed to lower PM_2.5_ environments in winter decreased by 40%, while those exposed to higher PM_2.5_ levels more than doubled. Such shifts in exposure risk show spatial congruity, particularly in cities in India and China, where regions with milder PM_2.5_ concentrations in summer transition to regions with higher concentrations in winter.

In the 120 global cities examined, the annual average O_3_ concentrations displayed a decline similar to the trends observed for PM_2.5_ concentrations. The O_3_ concentrations were noted to decrease from 120.51 μg/m^3^ in 2019 to 116.16 μg/m^3^ in 2021, further diminishing to 114.57 μg/m^3^ in 2022. During the study period, it was found that 50.8% of the cities under consideration exhibited a 4-year average O_3_ concentration below 100 μg/m^3^. These cities are predominantly located in regions such as the US (67.6 μg/m^3^), Canada (59.4 μg/m^3^), Australia (63.6 μg/m^3^), and European countries (71.6 μg/m^3^). On the other hand, a smaller portion, around 13.3%, registered a 4-year average O_3_ concentration of less than 60 μg/m^3^. In stark contrast, nearly half of the cities globally presented a 4-year average O_3_ concentration surpassing 100 μg/m^3^ over the research span. Such cities were chiefly located in India (231.8 μg/m^3^), China (169.5 μg/m^3^), Japan (121.5 μg/m^3^), and South Korea (136.2 μg/m^3^). Notably, northern Indian cities like Chennai (217.3 μg/m^3^) and Kolkata (265 μg/m^3^), as well as Chengdu (173 μg/m^3^) in central China, recorded O_3_ concentrations far exceeding the O_3_ threshold set by the WHO's AQG in 2021. In terms of the seasonal trends, globally, the highest proportion of cities exposed to high O_3_ concentrations (>100 μg/m^3^) occurred in summer, representing 19.2% of the selected cities, followed by spring (14.2%), autumn (12.5%), and winter (8.3%). Cities persistently exposed to heightened O_3_ environments exhibited distinct spatial clustering, primarily in central China and northeastern India.

In the key cities of focus (Fig. S1), Delhi registered the pinnacle 4-year average PM_2.5_ concentration at 161.89 ± 60.56 μg/m^3^, while Sydney recorded the nadir at 25.03 ± 7.64 μg/m^3^. In terms of seasonal variations, PM_2.5_ concentrations in cities such as Berlin, London, Tokyo, Seoul, and Beijing predominantly exhibited a winter > spring > autumn > summer sequence. In contrast, other cities displayed varied seasonal changes: cities like Rome and Sydney peaked in the spring and bottomed out in the summer, while Los Angeles witnessed its minimum concentrations in spring. The highest and lowest O_3_ concentrations were identified in Delhi and Rome, respectively, with values of 140.2 ± 37.89 and 25.34 ± 5.74 μg/m^3^. Following closely are Mexico City, Seoul, and Beijing, all of which have O_3_ concentrations surpassing 60 μg/m^3^. In contrast, other cities exhibit O_3_ levels ranging between 30 and 50 μg/m^3^. Additionally, it was observed that the peak O_3_ concentrations for these focal cities occurred in summer, while the lowest levels were typically registered in winter (except for Delhi, where the minimum levels were observed in autumn), aligning with the global seasonal variations in O_3_ concentrations.

### 3.2 Global characteristics of PM_2.5_ and O_3_ compound pollution

**Fig. 3:**
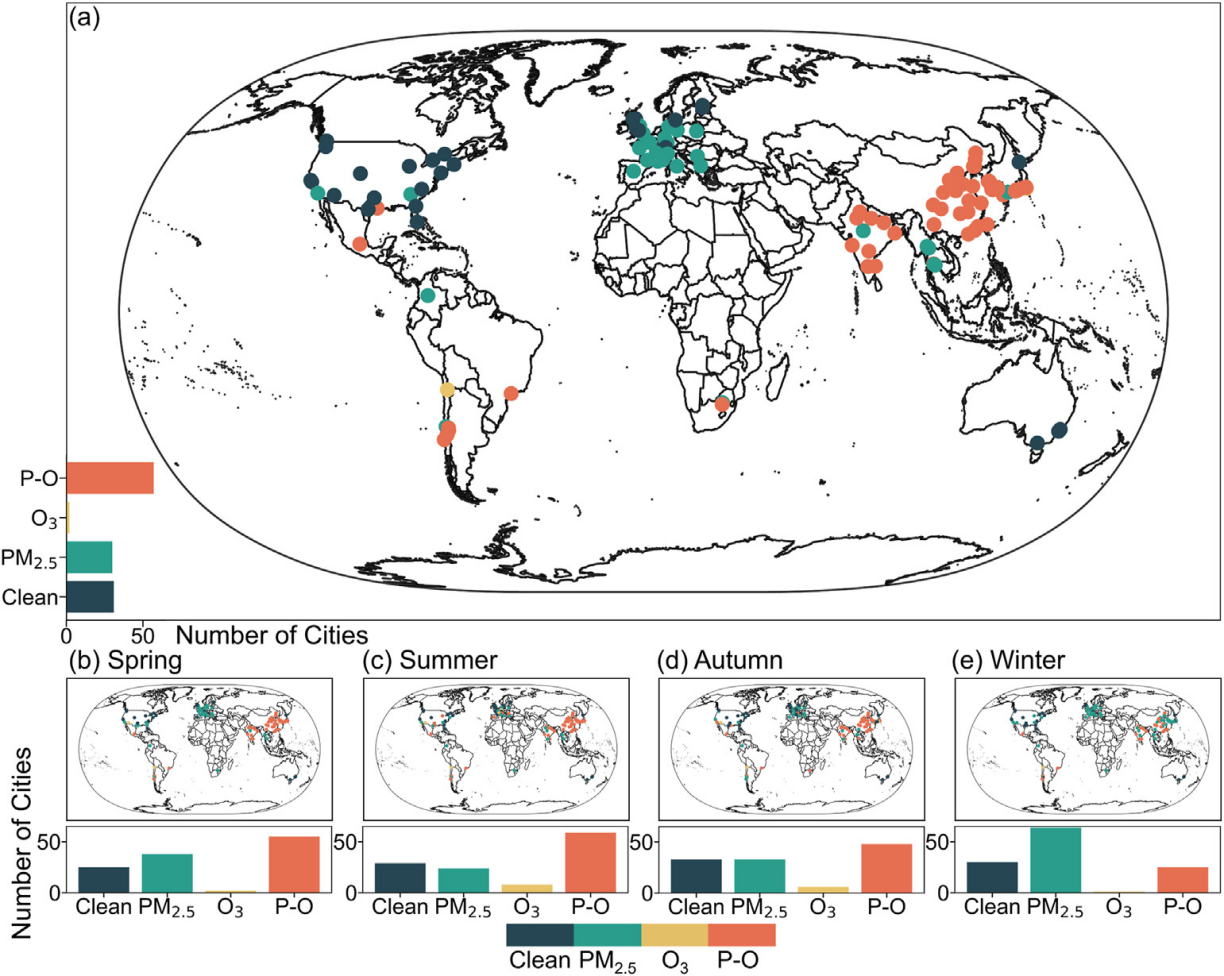
The spatial distribution (a) and seasonal variation (b–e) of PM_2.5_-O_3_ compound pollution conditions in 120 cities worldwide from 2019 to 2022.

While the PM_2.5_ concentrations in most global cities have yet to reach the thresholds set by AQG, urban O_3_ pollution is becoming increasingly severe. There's a noticeable trend of compound pollution involving both PM_2.5_ and O_3_ in various global regions. This subsection, based on the methodology provided in *Section 2.3*, offers a comprehensive analysis of the spatiotemporal variations in PM_2.5_ and O_3_ compound pollution across 120 global cities during the research period (Fig. 3). Spatial statistics reveal that only 25.8% of the studied cities enjoy a relatively unpolluted environment (Clean). A significant proportion of these cities reside in the US, representing approximately 82% of all US cities, with several others in Northern Europe. Conversely, almost half (47.5%) of the global cities evaluated were subjected to PM_2.5_-O_3_ compound pollution during the study timeframe. This form of pollution predominantly affected cities in countries such as Chile (4), China (22), South Korea (10), Japan (7), and India (9). Additionally, 25% of cities are exposed to a PM_2.5_ dominant polluted environment, predominantly found in Europe, accounting for roughly 67.9% of European cities (Fig. 3a). From a seasonal perspective, spring, summer, and autumn witness the peak periods for global PM_2.5_-O_3_ compound pollution. During these three seasons, an average of over 40% of cities experience PM_2.5_-O_3_ compound pollution. Notably, during the summer, almost 50% of the selected cities are exposed to PM_2.5_-O_3_ compound pollution. These cities are primarily clustered in South Korea, eastern and southern China, and northern India (Fig. 3b–e). In stark contrast, less than 25% of cities worldwide are exposed to PM_2.5_-O_3_ compound pollution in winter, such as Delhi and Mumbai in India, and Shijiazhuang and Chengdu in China. Conversely, during winter, 53.3% of global cities face PM_2.5_ dominant pollution. These cities were dispersed across various continents, with European nations and northern China marking significant regions for winter PM_2.5_ dominant pollution, for instance, cities like Rome (Italy), Paris (France), and Hamburg (Germany).

As global air pollution concerns intensify, countries worldwide have issued stringent air pollution control strategies based on their specific conditions, leading to a shift in the dominant forms of air pollution. For the first time, Fig. S2 reveals the spatial characteristics of changes in dominant air pollution types across 120 global cities from 2019 to 2022. Overall, there was a positive shift towards cleaner urban environments: cities classified under the “Clean” category rose from 24 in 2019 to 26 in 2021 and further rose to 31 in 2022. Meanwhile, cities dominated by PM_2.5_ pollution increased slightly from 37 in 2019 to 38 in 2021 but then saw a reduction to 33 by 2022. Furthermore, cities under the bracket of O_3_ dominant pollution never exceeded 5% of the analyzed cities throughout the study period. Spatially, our analysis found that 24 cities, such as Berlin and London in Europe, consistently showed PM_2.5_ as the dominant pollutant during the entire study timeframe. In stark contrast, 46 cities persistently witnessed PM_2.5_-O_3_ compound pollution. Key cities in this category include Shijiazhuang and Shenyang in China, as well as Delhi and Mumbai in India. Seven cities, primarily situated in parts of India and Europe, transitioned from being PM_2.5_-dominant to experiencing PM_2.5_-O_3_ compound pollution. Meanwhile, 11 cities, predominantly located in Japan, some European regions, and Chile (including Yokohama, Japan; Lyon, France; and Rancagua, Chile), shifted from the PM_2.5_-O_3_ compound pollution to either PM_2.5_ dominant pollution or O_3_ dominant pollution. Furthermore, eight cities, dispersed across regions like the US, Australia, and Germany (for example, Chicago, US; Sydney, Australia; and Wiesbaden, Germany), transitioned from either O_3_ or PM_2.5_ dominant pollution to a “Clean” classification within the research period.

### 3.3 Exposure risk assessment of compound pollution

**Fig. 4:**
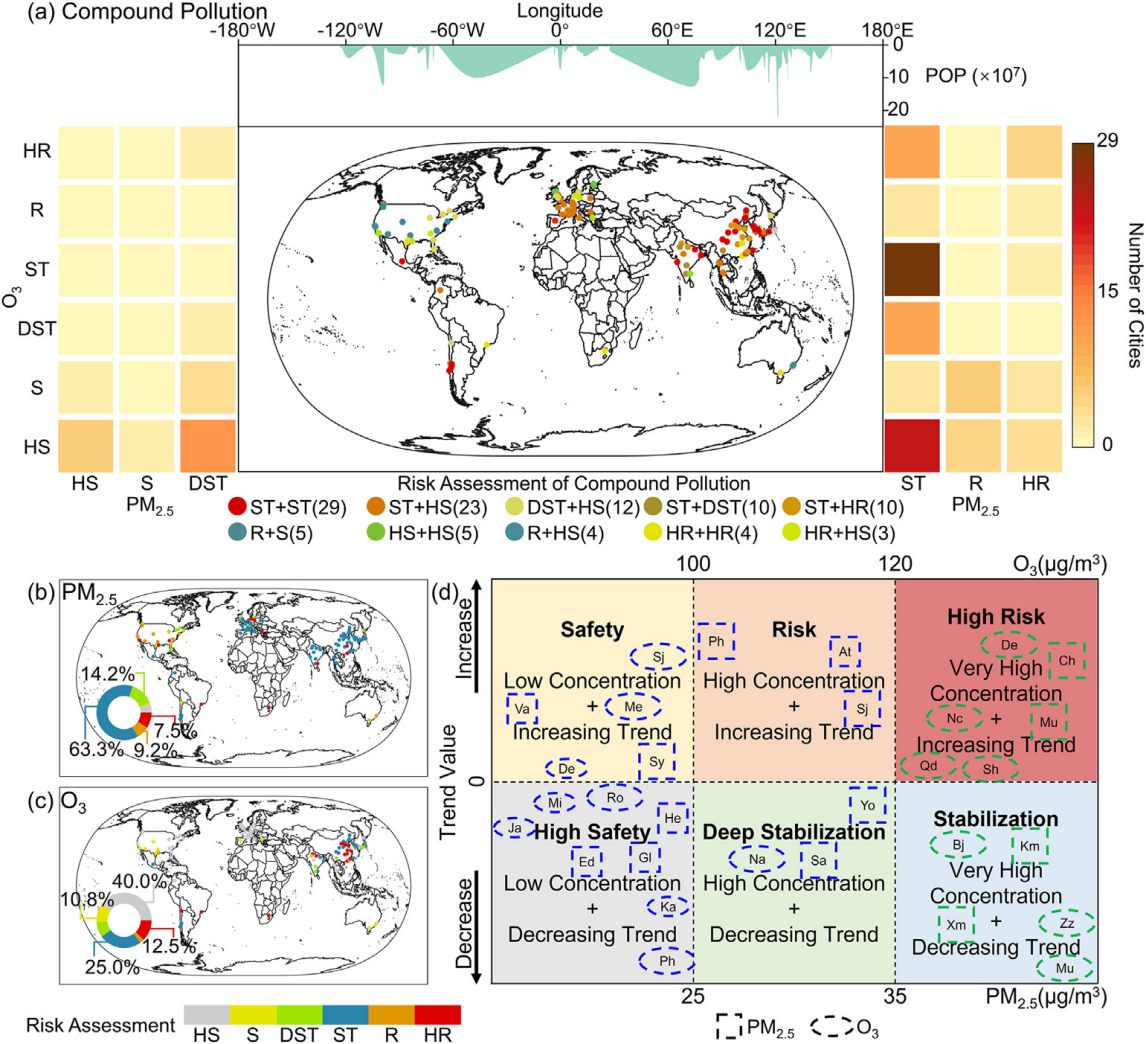
Exposure risk assessment of compound pollution across 120 cities from 2019 to 2022 (a); the line chart indicates the population count; the heatmap shows the number of city sites under different compound pollution exposure risks. PM_2.5_ and O_3_ pollution exposure risk assessment for 120 cities from 2019 to 2022 (b–c). Exposure risk assessment for select cities from 2019 to 2022 (d); blue borders represent cities in developed countries, and green borders indicate cities in developing countries. Smaller square or oval borders suggest a city population of less than 1 million (10^6^), while larger ones indicate the opposite.

The intensification of PM_2.5_ and O_3_ pollutants in the atmosphere presents diverse environmental exposure risks in cities globally. Fig. 4 illustrates the spatial distribution of air pollution exposure risks in 120 global cities during the study period. Our analysis identifies the primary types of compound pollution exposure risks in global cities as ST + ST, ST + HS, DST + HS, ST + DST, ST + HR, ST + DST, ST + HR, HS + HS, ST + HS, and DST + HS. Notably, ST + ST and ST + HS emerge as the most critical exposure risk types related to PM_2.5_-O_3_ compound pollution. Among the selected cities, 29 exhibit the ST + ST exposure risk type, predominantly located in China, South Korea, Japan, India, and Chile. Characterized by PM_2.5_ and O_3_ concentrations exceeding 35 μg/m^3^ and 100 μg/m^3^ respectively, these cities, though witnessing a declining trend, will subject their populations to significant compound pollution risks in the future. In contrast, 23 cities worldwide manifest the ST + HS compound pollution exposure risk type, mainly situated in Thailand, the UK, France, and Germany. Such cities, while presenting PM_2.5_ concentrations above 35 μg/m^3^ and O_3_ concentrations below 100 μg/m^3^, display a substantial decline in pollutant concentrations. Consequently, while they currently experience significant compound pollution risks, the consistent decline in O_3_ levels suggests a hopeful trajectory towards reduced risks. Additionally, 12, 10, and 10 cities globally show compound pollution exposure risks of DST + HS, ST + DST, and ST + HR, respectively. This includes Chicago, Boston, and Miami in the US, Shijiazhuang and Qingdao in China, and Delhi and Lucknow in India. In these cities, at least one of the PM_2.5_ or O_3_ concentrations falls below the AQG threshold and exhibits a continued declining trend, which results in a gradual reduction in compound pollution risks. From a demographic perspective, in densely populated Asian regions (over 200 million), the compound pollution risks are largely categorized into three types: ST + DST (10), ST + HR (10), and ST + ST (25). In Europe, a cumulative population exceeding 20 million is exposed to environments with compound pollution risk levels of HS + HS (5) and ST + HS (15). In North America, the predominant exposure risk is DST + HS (7).

The analysis of the individual trends in PM_2.5_ and O_3_ concentrations shows that approximately 63.3% (or 76) of cities worldwide are exposed to an environment with a PM_2.5_ risk type of ST. These cities are primarily located in Germany, France, China, India, South Korea, Japan, Thailand, and Chile, with examples including Tokyo (Japan), Busan (South Korea), Chongqing (China), Lucknow (India), Rome (Italy), Paris (France), and Santiago (Chile). Furthermore, cities with a PM_2.5_ exposure risk type of DST account for about 14.2% globally. They are predominantly found in the eastern and southern parts of the US, as well as in southeastern Canada, like Toronto (Canada) and Chicago (US). Moreover, it was noted that approximately 16.7% of the cities globally present PM_2.5_ exposure risks classified as R and HR. These cities, primarily located in the western US (including Phoenix and Philadelphia), display PM_2.5_ concentrations oscillating between 25 and 35 μg/m^3^ and exceeding 35 μg/m^3^, respectively, both indicating an increasing pattern. Additionally, a combined 5.8% of the cities, exemplified by Vancouver, exhibited PM_2.5_ exposure risks defined as HS and S, characterized by concentrations under 25 μg/m^3^. It's noteworthy that those within the ‘S’ classification reveal a rising PM_2.5_ trend (Fig. 4b). Regarding O_3_, over 50% of cities worldwide have O_3_ exposure risk types of HS and S. These cities are largely spread across the eastern US and most European regions, such as Berlin (Germany), London (UK), and Miami (US). Additionally, cities with O_3_ exposure risk types of ST or HR are primarily located in northern India, eastern China, South Korea, and Japan. Among these, cities with an exposure risk type of HR represent 12.5% and are chiefly centered in southeastern China, including cities like Shanghai and Jinan (Fig. 4c). From a combined perspective of population and economic levels, cities exposed to PM_2.5_ (or O_3_) concentrations below 35 μg/m^3^ (or 120 μg/m^3^) are largely found in developed countries. Examples are Chicago, San Antonio, Helsinki, and Sydney. About 125 million people in these areas can enjoy the reduced exposure risks brought by good air quality (low pollutant concentrations). In stark contrast, cities in developing nations like Beijing, Mumbai, and Delhi continue grappling with exacerbated pollutant concentrations. An estimated populace of 218 million endures heightened pollution environments, consequently intensifying their vulnerability to associated exposure risks.

### 3.4 Spatial association between PM_2.5_-O_3_ compound pollution and precursors

**Fig. 5:**
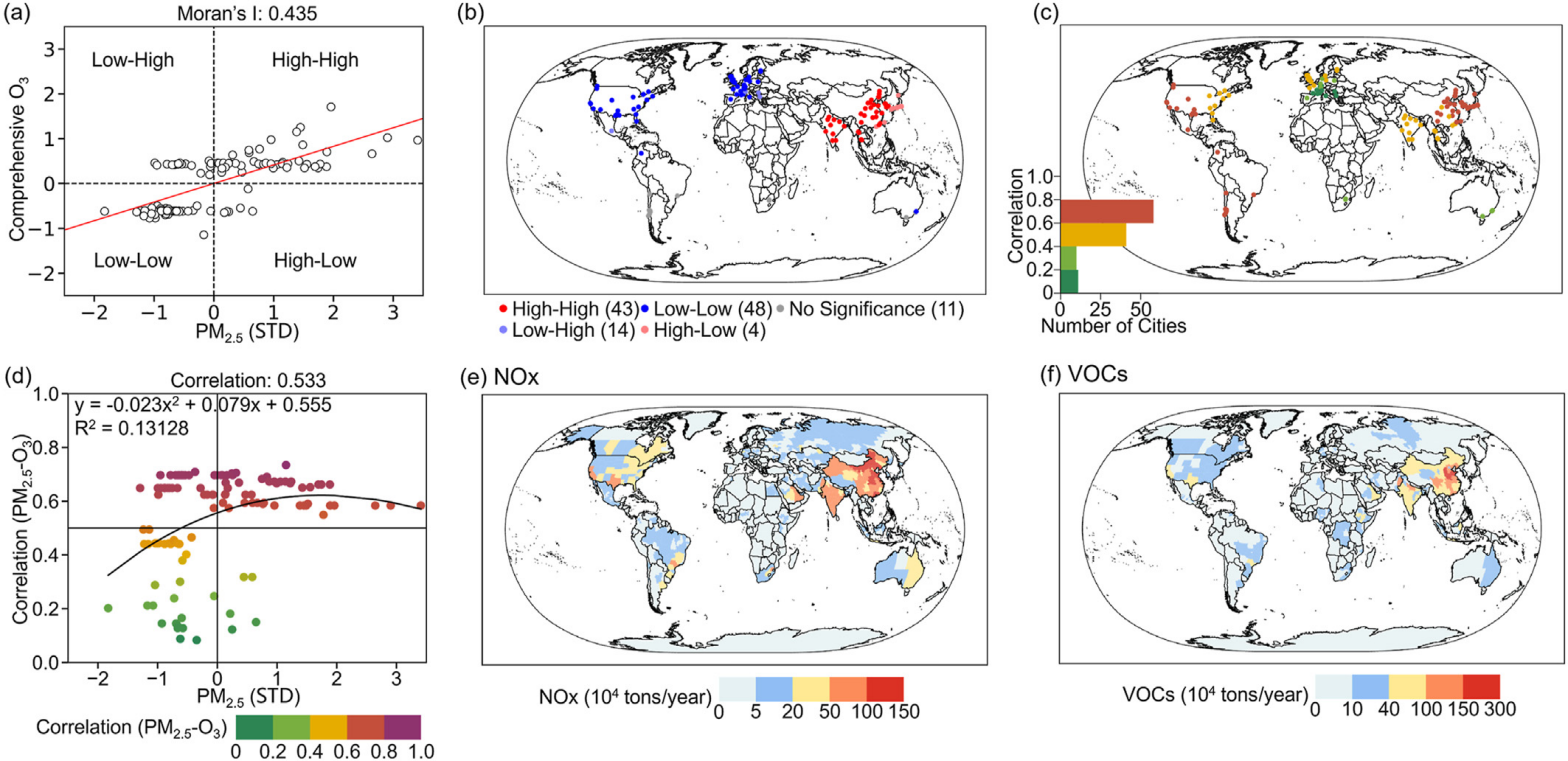
Distribution of PM_2.5_-O_3_ Bi-Moran's I and spatial distribution characteristics for 120 city sites from 2019 to 2022 (a–b); spatial distribution characteristics and trend features of the spatial correlation coefficient of PM_2.5_-O_3_ for 120 city sites from 2019 to 2022 (c–d); global spatial distribution of NOx and VOCs (e–f).

The environmental exposure risks caused by PM_2.5_-O_3_ compound pollution are increasingly severe. A quantitative elucidation of the spatial correlation between PM_2.5_ and O_3_ concentrations, along with their spatial association with precursors, holds paramount importance for devising coordinated emission reduction strategies for PM_2.5_ and O_3_ concentrations under forthcoming sustainable development paradigms. In Fig. 5a and b, the scatter plot of PM_2.5_-O_3_ bivariate Moran's I and the spatial clustering distribution for 120 global city stations are depicted. It can be observed that the bivariate Moran's I for PM_2.5_ and O_3_ is 0.435 (Moran's I > 0 indicates clustering), and it has passed the significance test (*P* < 0.05). Such results underscore a significant positive spatial correlation between PM_2.5_ and O_3_ concentrations. Specifically, 43 cities worldwide have their bivariate Moran's I for PM_2.5_ and O_3_ concentrations in the first quadrant, indicating a High–High spatial clustering pattern. Predominantly, these cities are located in regions such as China, India, South Korea, and Thailand, marked by high levels of both PM_2.5_ and O_3_ concentrations. Meanwhile, bivariate Moran's I for PM_2.5_ and O_3_ concentrations in 48 cities, mainly in the US, Canada, the UK, and France, were observed in the third quadrant, indicating a low–low spatial clustering pattern with low PM_2.5_ and O_3_ concentrations. In addition, certain cities in Japan and Mexico were ascertained to manifest either a low-high or high-low spatial clustering paradigm.

To further study the spatial association features between PM_2.5_ and O_3_ concentrations, we employed spatial correlation analysis methods to quantitatively reveal the correlation between PM_2.5_ and O_3_ concentrations (Fig. 5c). The results indicate that the correlation coefficient (Correlation) of PM_2.5_-O_3_ for the selected cities during the study period is all greater than zero, indicative of a positive correlation between PM_2.5_ and O_3_ concentrations. Specifically, in 58 cities located in eastern China, Japan, South Korea, the western US, and central Chile, the correlation coefficient of PM_2.5_ and O_3_ concentrations exceeded 0.6. In 41 cities in India, the UK, and the eastern US, this coefficient ranged between 0.4 and 0.6, such as in Delhi (0.584), Miami (0.443), and London (0.441). Additionally, fewer than 25 cities, predominantly in central and southern Europe, exhibited a Correlation below 0.4, with cities like Madrid and Zürich registering 0.3 and 0.202, respectively. Upon conducting multivariate regression analyses on PM_2.5_ and the correlation coefficient (R^2^ = 0.13128) (Fig. 5d), it was discerned that as PM_2.5_ concentration remained below 110 μg/m^3^ (STD: 1.717), the Correlation increased concomitant with the elevation of PM_2.5_ concentration. However, upon reaching a peak value of 0.623, the Correlation began to wane with increasing PM_2.5_ concentrations. These observations suggest that over 60% of cities worldwide exhibit a marked synergistic fluctuation between PM_2.5_ and O_3_ concentrations, underscoring the potential for coordinated management approaches in subsequent years. Significantly, through the analysis of the potential spatial associations between PM_2.5_ and O_3_ concentrations and their predominant precursors (NOx and VOCs) (Fig. 5e and f), it was ascertained that regions in China and India, characterized by elevated PM_2.5_ and O_3_ concentrations, also reported the highest emissions of NOx and VOCs, each exceeding an annual emission threshold of 1 million tons. Trailing them was the west coast of the US, with annual emissions of NOx and VOCs surpassing 500,000 tons. Such findings underscore the pivotal role that the cumulative emission effects of NOx and VOCs assume in shaping regional atmospheric pollution.

### 3.5 Potential for global coordinated management of PM_2.5_-O_3_ compound pollution

**Fig. 6:**
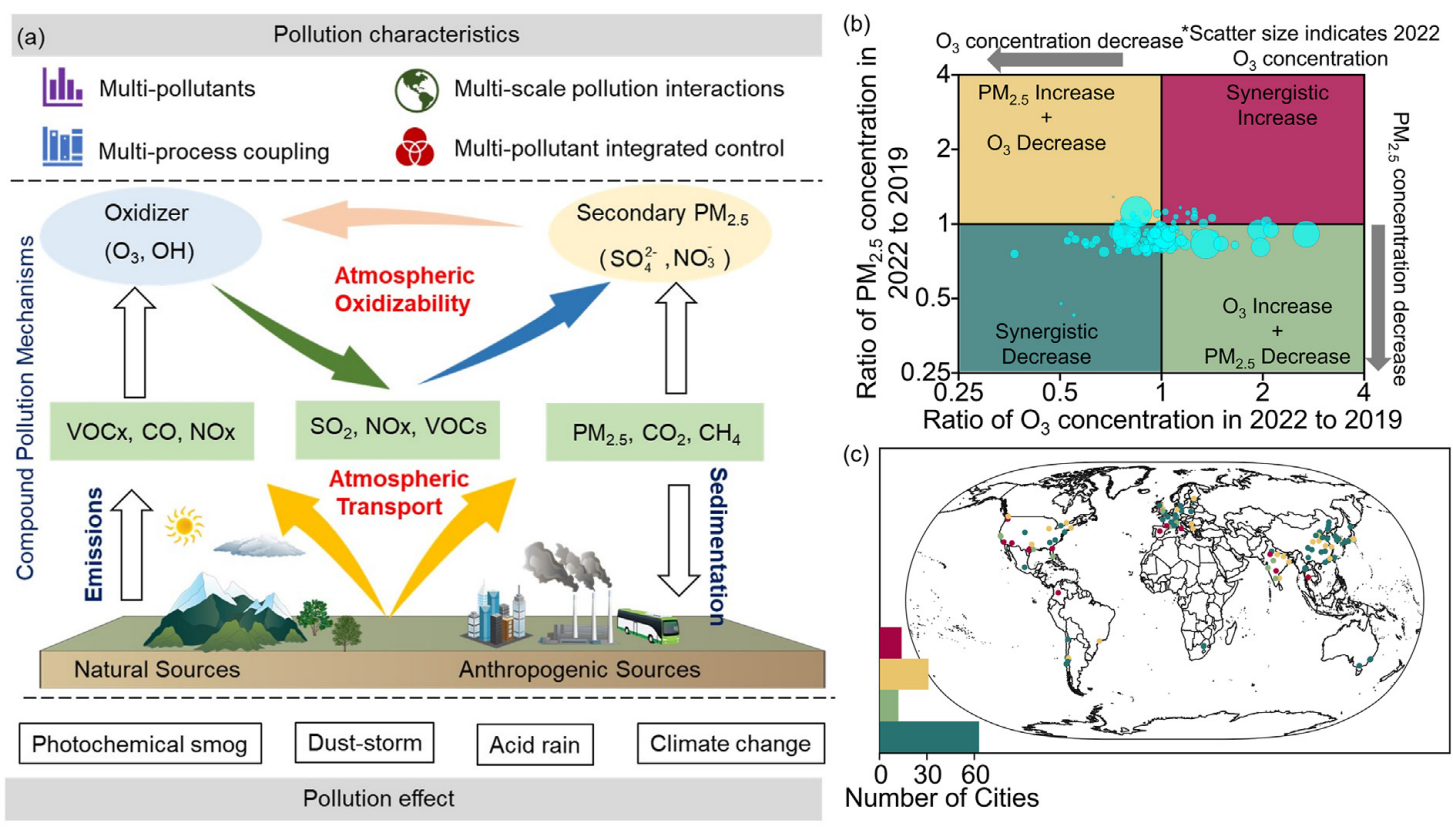
Mechanism features of PM_2.5_-O_3_ compound pollution (a), quadrant distribution of regional synergistic management potential (b), and spatial distribution (c). Specifically, (b) categorizes the variations in PM_2.5_ and O_3_ concentrations into the following four types based on their ratio: Synchronized increase of PM_2.5_ and O_3_ concentrations (First quadrant, both PM_2.5_ and O_3_ concentration ratios >1); increase in PM_2.5_ concentration with a decrease in O_3_ concentration (Second quadrant, PM_2.5_ concentration ratio >1 and O_3_ concentration ratio <1); synchronized decrease of PM_2.5_ and O_3_ concentrations (Third quadrant, both PM_2.5_ and O_3_ concentration ratios <1); decrease in PM_2.5_ concentration with an increase in O_3_ concentration (Fourth quadrant, PM_2.5_ concentration ratio <1 and O_3_ concentration ratio >1). The bar chart in (c) indicates the number of cities for each synergistic change type.

Based on the preceding sections, it can be conclusively deduced that PM_2.5_-O_3_ compound pollution manifests characteristics of overlapping pollution types, intertwined processes, and interactions across multiple scales. These distinct features serve as a robust scientific underpinning for the evaluation of potential coordinated management of PM_2.5_-O_3_ compound pollution, as depicted in Fig. 6a. In this segment, the potential was analyzed by examining the ratio of annual average concentration changes of PM_2.5_ and O_3_ between 2019 and 2022 across 120 global cities (Fig. 6b and c). Statistical results indicate that between 2019 and 2022, 63 cities achieved a coordinated decline in the annual average concentrations of PM_2.5_ and O_3_, representing 52.5% of the total cities studied. These cities registered an average decrease of 13.97% in PM_2.5_ and 19.18% in O_3_ concentrations. Geographically, a majority of these cities are situated in China (16), South Korea (8), and Japan (7). In contrast, 14 cities, representing 11.67% of the total, experienced a concurrent augmentation in the annual average concentrations of PM_2.5_ and O_3_ during the assessment period. Their average concentrations surged by 6.17% and 23.99%, respectively. Predominantly, these cities are located in the US (6) and India (2). Furthermore, a seesaw effect—characterized by a decrease in PM_2.5_ concentration concurrent with an increase in O_3_ concentration, or vice versa—was observed in the annual average concentrations of PM_2.5_ and O_3_ in 43 cities throughout the study's duration. These cities spanned diverse global locations, with the Asian region (20) exhibiting the most marked seesaw effect.

## 4 Discussion

### 4.1 PM_2.5_ and O_3_ compound pollution and synergistic control of spatial heterogeneity

The cities with frequent PM_2.5_ and O_3_ compound pollution are mainly in the Asian region, especially in China and India, where the number of compound pollution episodes is higher than in other regions, and the exposure risk of PM_2.5_ and O_3_ compound pollution was at the ST + ST level during the study period. One of the most important reasons for this is the high-speed economic development that has led to significant anthropogenic emissions, particularly of VOCs and NOx, which are precursors that promote O_3_ production [[51], [52], [53]]. For instance, Beijing and the Pearl River Delta in China effectively controlled particulate matter pollution, represented by PM_2.5_, after the implementation of the Action Plan for the Prevention and Control of Air Pollution. However, compound pollution with high concentrations of PM_2.5_ and O_3_ has become the main problem nowadays. There are multiple reasons contributing to this change, but the fundamental reason is the higher emission intensity in these regions, while the meteorological conditions have been more favorable for O_3_ generation in recent years [[54], [55], [56], [57], [58]]. Furthermore, at the O_3_ chemistry level, this phenomenon occurs because the effects of precursors NOx and VOCs are not linear, and O_3_ concentrations may rebound as NOx emissions are reduced [[59], [60], [61]]. Similarly, the main reason for the sharp increase in O_3_ concentrations in India in recent years is closely related to the emission of O_3_ precursors. According to Chen et al. [62], reducing NOx emissions by 50% in India in 2018 resulted in a 10%–50% increase in O_3_. Conversely, a 50% reduction in VOC emissions can lead to a 60% reduction in O_3_. In 2019, India's annual average PM_2.5_ concentration was 91.7 μg/m^3^, which is still higher than the WHO IT-1 (35 μg/m^3^) [63]. This means that while India has not yet met the PM_2.5_ standard, O_3_ pollution has increased, resulting in more compound pollution events.

Compared to Asian cities, European and North American cities have relatively low levels of PM_2.5_ and O_3_ compound pollution. This pollution is mainly dominated by either PM_2.5_ or O_3_, and most of the population exposure risk status is DST + HS and ST + HS. The industrial structure of most cities in Europe and North America is dominated by tertiary and emerging industries, which are most notably characterized by low emissions and high returns. Compared to most Asian cities that are still reliant on secondary industries, Europe and North America have lower levels of industrial emissions, which means that PM_2.5_ concentrations are also significantly lower than in Asian cities [8,[64], [65], [66]]. Some North American cities have PM_2.5_ concentrations that reach the AQG levels set by the WHO. Unfortunately, increased emissions of ozone precursors and unfavorable meteorological factors have led to O_3_ pollution becoming a new challenge to atmospheric pollution in some North American and European cities [67,68]. Equally important, air pollution in the eastern US and southern Europe has been worsened by wildfires and cross-border pollutant transport, which has serious implications for the health of regional populations [69,70].

Analysis of the characterization of synergistic emissions of PM_2.5_ and O_3_ reveals that there is a significant positive spatial correlation between global PM_2.5_ and O_3_ concentrations. This relationship is mainly determined by the homology of PM_2.5_ and O_3_ concentrations. Previous studies have shown that PM_2.5_ precursors include SO_2_, NOx, NH_3_, VOCs, and primary PM_2.5_. Among these, NOx and VOCs are the most significant precursors in O_3_ chemistry [71,72]. At the same time, we find significant spatial consistency between the spatial and temporal patterns of global NOx and VOC emissions and the associated strengths of PM_2.5_ and O_3_ concentrations. In other words, regions with stronger spatial correlation between PM_2.5_ and O_3_ concentrations have higher emissions of NOx and VOCs, further suggesting that synergistic emission reduction of NOx and VOCs is key to achieving synergistic control of PM_2.5_ and O_3_, for example, pollutants such as VOCs, NOx, etc. can be reacted into other compounds by electrocatalysis and thermal catalysis [73,74]. From the characteristics of synergistic changes in PM_2.5_ and O_3_ concentrations, 52.5% of the selected cities showed synergistic decreases. These cities are mainly located in East and South Asia. Appropriate adjustment of industrial layout in the future will greatly reduce the trend of PM_2.5_ and O_3_ compound pollution in these cities and realize sustainable development.

### 4.2 PM_2.5_-O_3_ correlation analysis of key cities

Through correlation analysis, we found high correlation areas and seasonal characteristics of PM_2.5_ and O_3_ concentrations. In Asian cities, particularly in East and South Asia, the interactions are greater because the static weather conditions in winter caused by Siberian high pressure often reduce vertical mixing in the atmosphere, leading to a build-up of pollutants close to the ground [75,76]. In addition, high summer temperatures and intense solar radiation provide favorable conditions for O_3_ formation in the tropical and subtropical regions of Asia. However, in monsoon climates, increased rainfall may wash out atmospheric pollutants, including precursors of O_3_. Previous studies have identified wind speed and shortwave radiation as the primary factors contributing to the fluctuations in PM_2.5_ concentrations in the Beijing area [77,78]. For O_3_, temperature is the most important correlation factor that affects its change [79]. Furthermore, based on research into air pollution mechanisms, it has been discovered that there is a strong positive correlation between PM_2.5_ concentration and extinction coefficient. Additionally, carbon-containing aerosols, which are one of the main components of aerosols, can also absorb light [80,81]. Therefore, areas with high concentrations of PM_2.5_, meaning high levels of atmospheric aerosols, will have a greater impact on local light intensity and, consequently, on the local production of O_3_ [9,82]. Previous studies have indicated that PM_2.5_ and O_3_ concentrations in Delhi exhibit distinct seasonal trends, with differences between summer and winter. Therefore, it is recommended to analyze them separately on a seasonal basis. During winter, high concentrations of PM_2.5_ have a significant impact on incident solar radiation, which affects O_3_ concentrations. In summer, PM_2.5_ is diluted due to ventilation effects, but O_3_ concentrations increase due to atmospheric oxidation [[83], [84], [85]]. In contrast, while Tokyo and Seoul have significantly better environmental levels than most Chinese and Indian cities, they still fall short of meeting WHO standards. The chemical industry and combustion source sectors in Japan have a significant impact on local VOC emissions, which indirectly contribute to local PM_2.5_ and O_3_ pollution [86]. The establishment of a “Road Transport” department in Seoul has led to an increase in the number of registered vehicles and kilometers driven, resulting in increased local PM_2.5_ and O_3_ pollution [87].

Air pollution is generally less problematic in Europe than in Asia due to milder climatic conditions and better atmospheric dispersion. However, seasonal peaks in PM_2.5_ occur during the winter months due to increased heating demand. Moderate high temperatures in Europe promote the formation of O_3_. However, extensive environmental policies and emission controls have reduced O_3_ precursor emissions, aiding in the regulation of O_3_ levels. In general, the more moderate changes in PM_2.5_ and O_3_ concentrations in the European region and the observed positive correlation between PM_2.5_ and O_3_ concentrations may be due to the decisive role of secondary photochemical processes in the formation of secondary particulate matter, especially in the absence of anthropogenic sources [88]. Previous studies have shown that the most significant sources of O_3_ and PM_2.5_ in London, Berlin, and Rome are boundary conditions, transport, biological emissions, and heating systems in winter [89,90]. For London, the most significant non-road transport emissions are likely from shipping activities in the English Channel [91].

In North America, industrial activities and automobile use are significant sources of PM_2.5_, particularly in urban and industrially dense areas. However, environmental regulations and policies, such as the Clean Air Act, help to control PM_2.5_ emissions. In addition, the transportation of pollutants across borders and high local ambient temperatures may exacerbate environmental pollution [92]. Environmental studies have reported that the composition of PM_2.5_ varies in areas of different dimensions due to factors such as geographical and climatic conditions, socio-economic status, and local industrial emissions [93,94]. These differences in PM_2.5_ composition may affect the interaction between PM_2.5_ and O_3_ [95]. Although Los Angeles is considered to be one of the most polluted areas in the US, its pollution levels are still lower than those of many cities in Asia [96]. Stricter emission standards have effectively controlled VOCs and NOx emissions in Los Angeles by reducing motor vehicle emissions, including petrol evaporation [[97], [98], [99]]. Mexico City has successfully reduced primary pollutant emissions over the past few decades. However, it still faces challenges in reducing secondary pollutant emissions, such as PM_2.5_ [100]. Previous studies have shown that the main reason for high local levels of O_3_ and PM_2.5_ during the outbreak closure was air quality exchange through valley passages. Domestic heating is a major contributor to local PM_2.5_ pollution, and increased solar radiation and household activities also contribute to O_3_ pollution [101].

Previous studies have shown that reducing emissions from wood heaters and power stations in the Sydney area can extend the life expectancy of the local population and have a positive impact on the local economy [102]. Sydney has experienced mild temperatures and meteorological conditions throughout the year. However, due to the intensification of the heat island cycle and the enhancement of urban roughness, there has been a heightened correlation between temperature and wind speed on local O_3_ concentrations. Additionally, there has been a high frequency of O_3_ and PM_2.5_ pollution extremes that are strongly correlated with the worsening of local hill fire events [103].

### 4.3 Policy and recommendations

In this study, we reveal the dynamic change characteristics of global PM_2.5_ and O_3_ compound pollution, exposure risk level, spatial clustering characteristics, and synergistic change rules, and propose the following policies and recommendations for global PM_2.5_ and O_3_ pollution treatment.

(1) As implications for future air pollution mitigation strategies, developed cities are advised to prioritize preventive pollution measures, ensuring the curtailment of high pollution incidents potentially triggered by unfavorable meteorological conditions or human-induced emissions. On the contrary, for cities in developing countries, like Delhi in India and the Beijing-Tianjin-Hebei region in China, it's imperative to draft strict air pollution control policies while placing emphasis on regional economic growth. Simultaneously, there should be proactive promotion of the green transformation of traditional industries, aiming to minimize industrial emissions, residential emissions, and transport-related emissions resulting from the growth of conventional industries.

(2) To meet the stipulated benchmarks for PM_2.5_ and O_3_, regions severely affected by compound pollution (such as China and India) should focus on strengthening end-point control measures in the industrial and transportation sectors, emphasize adjusting the industrial structure and substituting sources for processes like petrochemicals, industrial painting, and wood furniture, and optimize the energy structure of motor vehicles.

(3) Broadly speaking, in order to address the inequalities in air pollution exposure and associated risks, governmental departments across countries should actively explore spatial variations of air pollution exposure inequalities and their potential determinants under the 2030 United Nations Sustainable Development Goals. Economic development, income levels, industrial adjustments, education standards, and racial considerations should be incorporated into regional and national environmental health plans. It is vital to synchronize regional air pollution interventions with enhancements in healthcare. Addressing the challenges of unequal air pollution exposure is integral to forging a sustainable society.

(4) For regions achieving a coordinated decrease in PM_2.5_ and O_3_, local governmental departments should further refine the implementation plans for synergistic management of air pollutants and establish robust mechanisms to prevent a resurgence of PM_2.5_-O_3_ compound pollution events. For areas witnessing synchronized increases in PM_2.5_ and O_3_ concentrations, we recommend initially constructing high temporal and spatial resolution regional emission inventories, understanding the pollution mechanisms and potential sources of PM_2.5_ and O_3_ from atmospheric chemistry and regional transmission perspectives, and formulating targeted pollution reduction policies based on these findings.

Overall, such initiatives are crucial for promoting both high-quality ecological protection and high-quality economic development collaboratively.

### 4.4 Research limitations and prospects

This study has some limitations. It focuses on a short-term period from 2019 to 2022 to analyze the trends of PM_2.5_ and O_3_ concentrations. Typically, a 10-year time series is considered sufficient to assess short-term changes in air pollution levels, attributing observed fluctuations predominantly to changes in emissions rather than meteorological variations. The decision to focus on a shorter timeframe in this study is primarily driven by the emergent nature of PM_2.5_-O_3_ compound pollution challenges and the urgency in addressing them. However, this approach does bear limitations. The relatively brief period may not fully encapsulate the broader impacts of long-term meteorological patterns and emission change trends on air quality. As such, the findings presented herein should be interpreted with caution, acknowledging the potential for meteorological variations to influence the observed pollution levels over this period. To mitigate these limitations, this study incorporates a review of existing literature and attempts to contextualize the findings within the broader scope of ongoing research in the field of air quality and pollution control. By highlighting these limitations, the study aims to provide a transparent and critical assessment of its findings, contributing to the ongoing discourse on effective strategies for PM_2.5_ and O_3_ pollution management and encouraging further research that addresses these emerging challenges with a longer temporal analysis.

Furthermore, we will expand the temporal scope of our analysis by incorporating longer time series data. This will enable us to more accurately identify the underlying trends in PM_2.5_ and O_3_ pollution. Future research will seek to disentangle the respective contributions of changes in emissions and meteorological forcing over longer periods, thereby deepening our understanding of the dynamics governing air quality. Exploring the effectiveness of pollution control strategies across different meteorological and geographical contexts is crucial for developing more nuanced and effective approaches to air pollution management.

## 5 Conclusions

During the study period, globally, 30% and 50% of cities were exposed to high PM_2.5_ (>70 μg/m^3^) and O_3_ (>100 μg/m^3^) concentrations respectively. Elevated concentrations of PM_2.5_ and O_3_ were predominantly observed in cities of developing nations, notably China and India. Furthermore, it was noted that over 80% of global cities encountered peak PM_2.5_ values during winter, whereas peak O_3_ values were predominantly identified during summer months. Nearly 50% of cities worldwide were affected by PM_2.5_-O_3_ compound pollution. Countries like China, South Korea, Japan, and India suffered the most severe impacts from PM_2.5_-O_3_ compound pollution. With the exacerbation of O_3_ pollution from 2019 to 2022, it was observed that 44.2% of cities globally transitioned from being primarily affected by PM_2.5_ or other contaminants to a predominant influence of PM_2.5_-O_3_ compound pollution. Over 40 cities were identified as areas of high exposure risk to this compound pollution, with exposure risk types classified as Stabilization + Stabilization (29), Stabilization + High Risk (10), and High Risk + High Risk (4). From the perspective of regional economic levels, there is an inequality in exposure risk due to PM_2.5_-O_3_ compound pollution. Specifically, cities in developing nations were found to be at higher risk compared to their counterparts in developed countries. Between 2019 and 2022, 52.5% of cities worldwide achieved a coordinated decline in the annual average concentrations of PM_2.5_ and O_3_. These cities witnessed an average drop of 13.97% for PM_2.5_ and 19.18% for O_3_ concentrations. Notably, there was a significant spatial clustering characteristic in the concentrations of PM_2.5_ and O_3_ in these cities, accompanied by a positive spatial correlation. Additionally, nearly 12% of cities saw a synchronized increase in the annual average concentrations of PM_2.5_ and O_3_.

## CRediT authorship contribution statement

C.H.: supervision, conceptualization, writing–original draft, writing–review editing; J.H.L.: visualization, writing–original draft; Y.Q.Z.: data curation, resources; J.W.Z.: software; L.Z.: methodology; Y.F.W.: Investigation; L.L., S.P.: supervision.

## Declaration of competing interest

The authors declare that they have no known competing financial interests or personal relationships that could have appeared to influence the work reported in this paper.
