# Supplementary material for: Synergistic PM2.5 and O3 control to address the emerging global PM2.5-O3 compound pollution challenges
Source: Eco Environ Health. 2024 Apr 19;3(3):325–37. doi: 10.1016/j.eehl.2024.04.004 (PMC11400616; doi:10.1016/j.eehl.2024.04.004)
Supplement: Multimedia component 1 [file mmc1.docx]

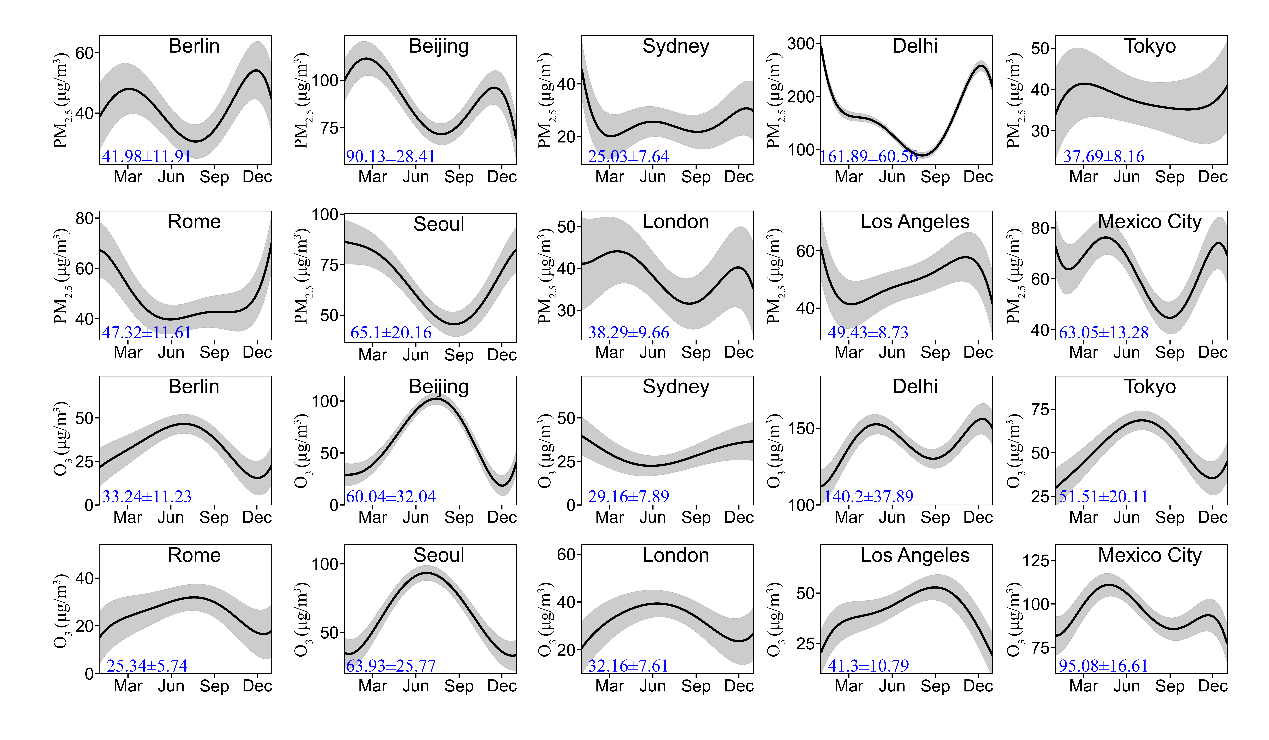


**Fig. S1.** Daily average PM_2.5_ and O_3_ concentration trends in key cities.


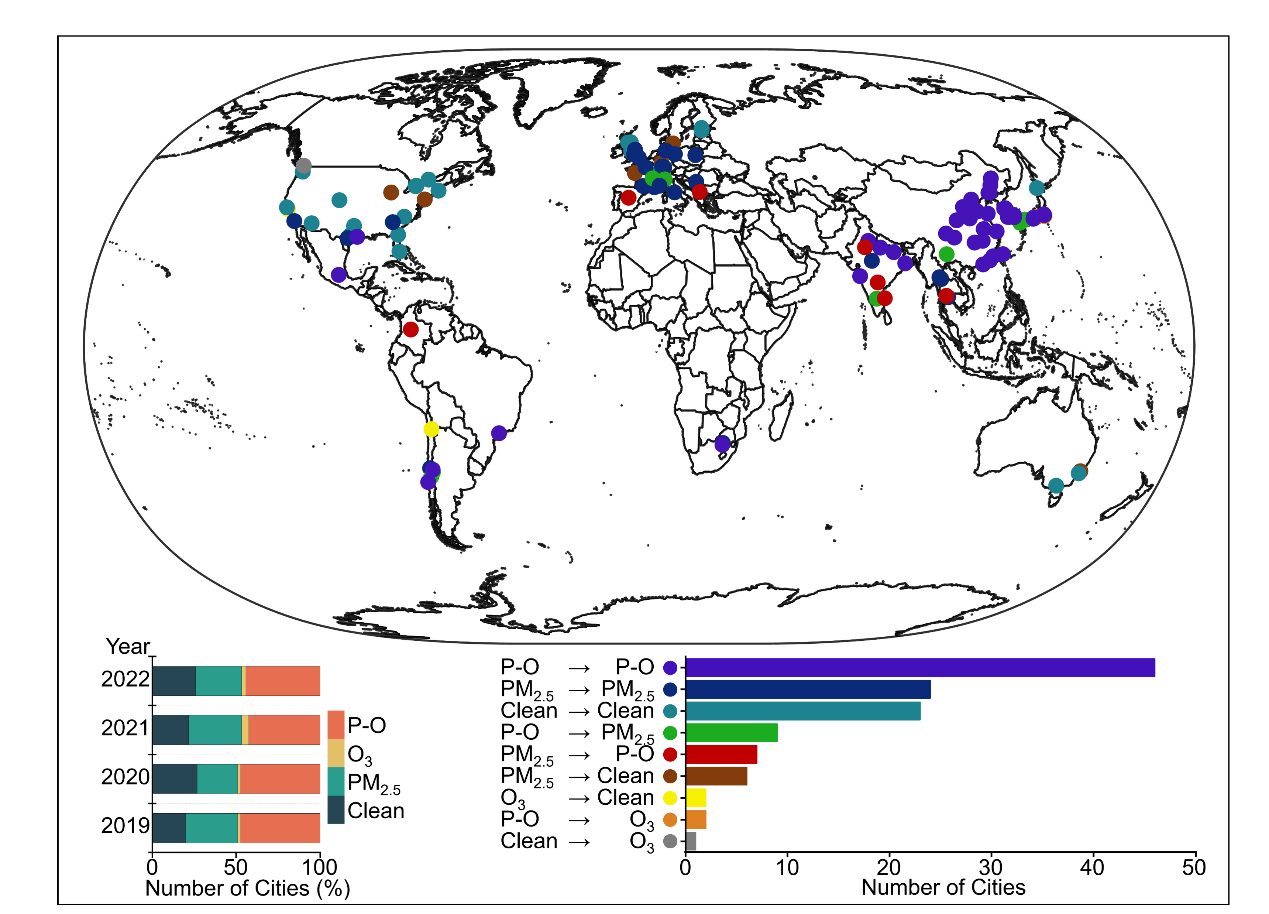


**Fig. S2.** From 2019 to 2022, the spatial distribution and phased variation characteristics of PM_2.5_-O_3_ compound pollution in 120 cities worldwide.
